# Supplementary material for: Complex‐centric proteome profiling by SEC‐SWATH‐MS
Source: Mol Syst Biol. 2019 Jan 14;15(1):e8438. doi: 10.15252/msb.20188438 (PMC6346213; doi:10.15252/msb.20188438)
Supplement: Supplementary file 8 — Dataset EV7 [file MSB-15-e8438-s008.zip › feature_plots_string/Q9H6R4.pdf]

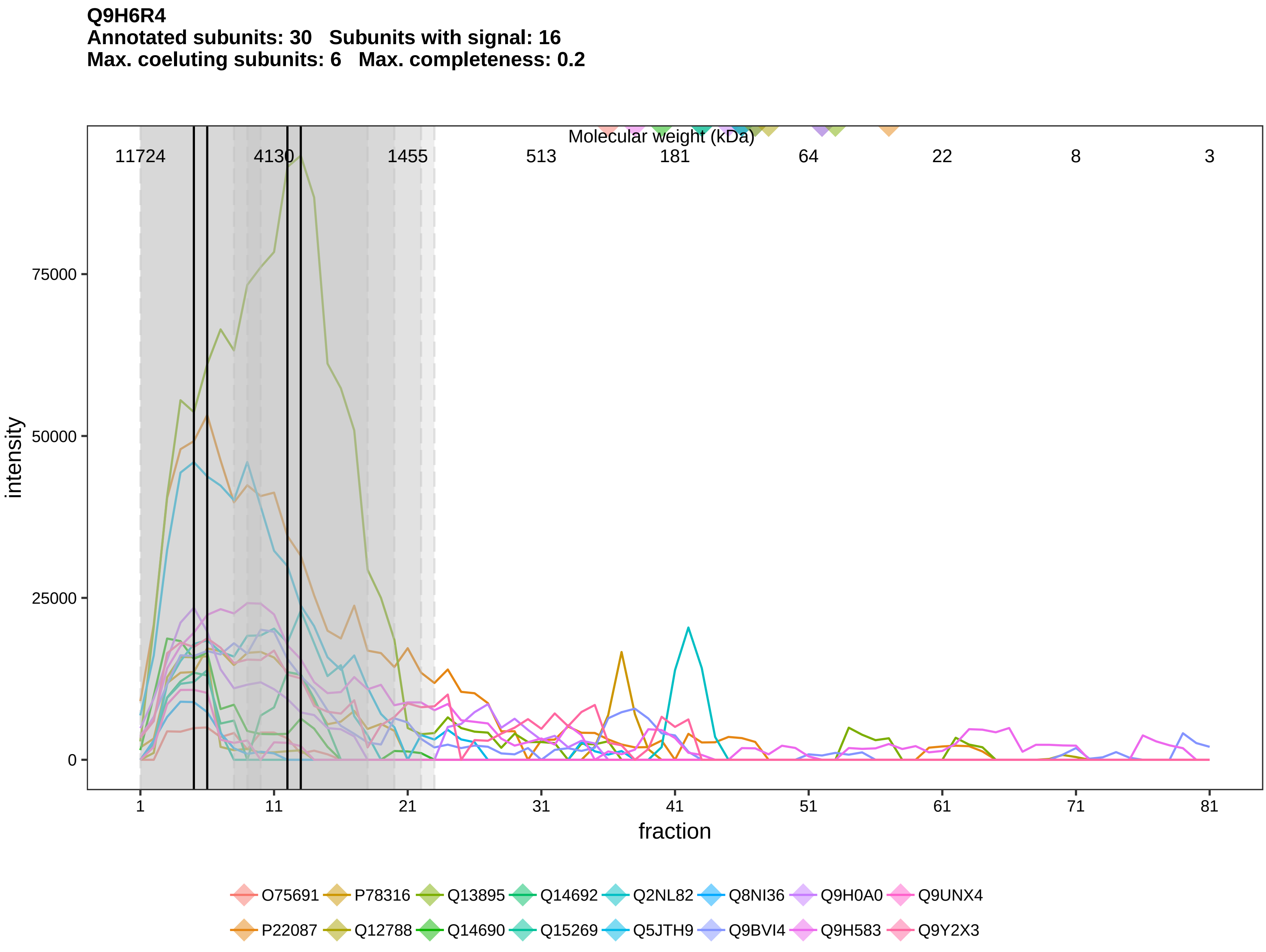
Q9H6R4
Annotated subunits: 30 Subunits with signal: 16
Max. coeluting subunits: 6 Max. completeness: 0.2
Molecular weight (kDa)
11724
4130
1455
513
181
64
22
8
3
intensity
75000
50000
25000
0
1
11
21
31
41
51
61
71
81
fraction
O75691
P78316
Q13895
Q14692
Q2NL82
Q8NI36
Q9H0A0
Q9UNX4
P22087
Q12788
Q14690
Q15269
Q5JTH9
Q9BVI4
Q9H583
Q9Y2X3
